# Endovenous Laser Ablation for Treatment of a Partially Thrombosed Proximal Great Saphenous Vein

**DOI:** 10.1155/2019/1726978

**Published:** 2019-06-12

**Authors:** Luca Spinedi, Hans Stricker, Daniel Staub, Heiko Uthoff

**Affiliations:** ^1^Division of Angiology, Ospedale Regionale di Locarno, Locarno, Switzerland; ^2^Division of Angiology, University Hospital Basel, University of Basel, Basel, Switzerland; ^3^Gefässpraxis am See, Hirslanden Clinic St. Anna, Lucerne, Switzerland

## Abstract

**Introduction:**

Superficial vein thrombosis of the great saphenous vein near to the saphenofemoral junction is generally treated with anticoagulation or surgically.

**Report:**

We present the case of a 70-year-old man with varicosities and a partially thrombosed great saphenous vein near to the saphenofemoral junction, treated with endovenous laser ablation of the great saphenous vein.

**Discussion:**

The case illustrates an alternative treatment option for superficial vein thrombosis of the great saphenous vein, which permits avoiding a prolonged anticoagulation or surgical procedure.

## 1. Introduction

Different treatment modalities ranging from anticoagulation to surgery have been suggested and are performed to treat a superficial vein thrombosis (SVT) of the great saphenous vein (GSV) [1]. In this case report, we present a case of a thrombotic mass located in the GSV near to the saphenofemoral junction (SFJ), treated by endovenous laser ablation (EVLA).

## 2. Case Presentation

**Figure 1:**
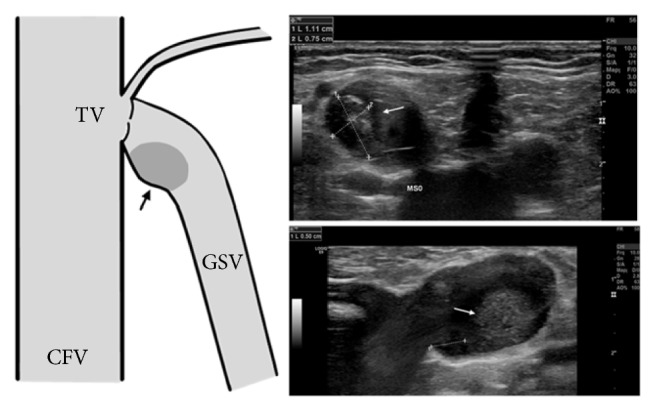
Partially obstructing, isoechogenic mass with hyperechogenic components (arrows) at the saphenofemoral junction in a medially situated eccentric dilatation of the great saphenous vein distally from the terminal valve.

A 70-year-old man was evaluated for a long history of symptomatic varicose veins (pain and perimalleolar edema formation) on his left leg. Except for a dyslipidemia and arterial hypertension treated, respectively, with simvastatin and lisinopril, no other comorbidities were known. Personal and family history for prior venous thromboembolic events or neoplasia were negative. The physical examination revealed varicose veins in the area of the knee and lower leg mediodorsally, with perimalleolar edema on the left side. No varicose veins or local signs of inflammation were present in the groin. On the duplex ultrasound examination, an insufficient left GSV was disclosed, showing a long-lasting reflux (>2sec.) from the SFJ down to the ankle. The maximum diameter of the vein was 10mm in the thigh. Unexpectedly, a 10x10x8 mm isoechogenic mass with an hyperechogenic component could be detected in the region of the SFJ, in an eccentric dilated segment of the GSV adherent both to the terminal valve and to the vein wall. This mass was only partially obstructing the GSF (Figure 1). No color Doppler signals could be detected within the structure. The deep venous system of the left leg was patent and sufficient, without postthrombotic sequelae. Several characteristics, such as the location in a dilated segment of the vein, the echogenicity, and the absence of a Doppler signal within the mass, favored a diagnosis of focal thrombosis.

**Figure 2:**
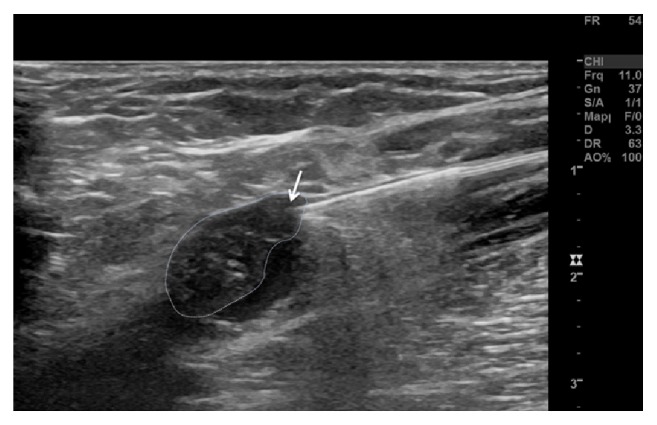
The tip of the laser fiber (white arrow) is stuck at the distal thrombotic mass (white-dashed line).

**Figure 3:**
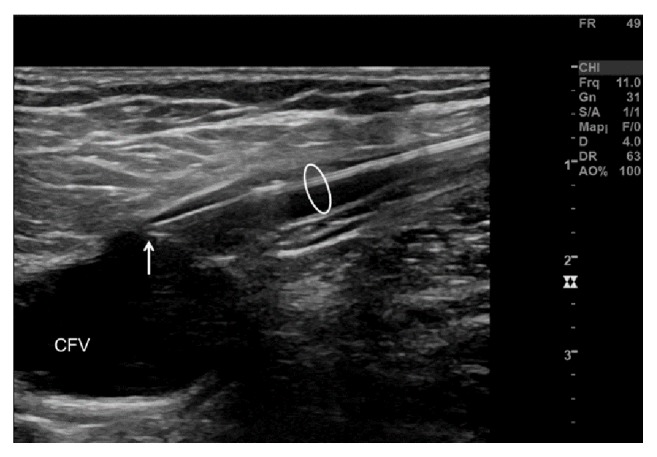
The tip of the laser fiber (arrow) is placed at the confluence between the great saphenous vein (white circle) and the common femoral vein (CFV).

We started a thromboembolic prophylaxis with Rivaroxaban 10mg once daily. In addition, we discussed with the patient an accelerated EVLA of the GSV with the aim to treat the varicose veins, excluding at the same time the thrombotic mass. The outpatient-based intervention took place 7 days later. At that time, the thrombotic mass was still present and unchanged. A 1470nm wavelength radial fiber with a diameter of 600*μ*m (ELVeS Biolitec Radial Laser) was inserted via a 21G introducer under echographic guidance from the mid third of the lower leg and pushed up to the SFJ. The thrombotic mass was passed under echographic guidance by maneuvering the tip laterally to the thrombotic mass (Figure 2) without dislodging the thrombus (Figure 3) by gently steering the fiber, applying distal external manual pressure onto the laser fiber. The laser fiber was eventually placed at the confluence of the GSV with the common femoral vein without a safety distance in order to exclude the thrombotic mass. After tumescence anesthesia with diluted prilocaine and epinephrine, EVLA was performed (10W/100J proximally and 8W/80J distally, for a total energy of 6614 Joules and a treated length of 66cm). Subsequently, the tributaries were treated by miniphlebectomy. At the end of the procedure, an extrinsic compression with gauzes and elastic compression stocking (23-32 mmHg) were applied. Prophylactic anticoagulation with Rivaroxaban 10mg daily was continued for 5 days, according to our internal protocol after standard EVLA. Wound healing occurred without relevant complications, and the patient was asymptomatic during the follow-up.

**Figure 4:**
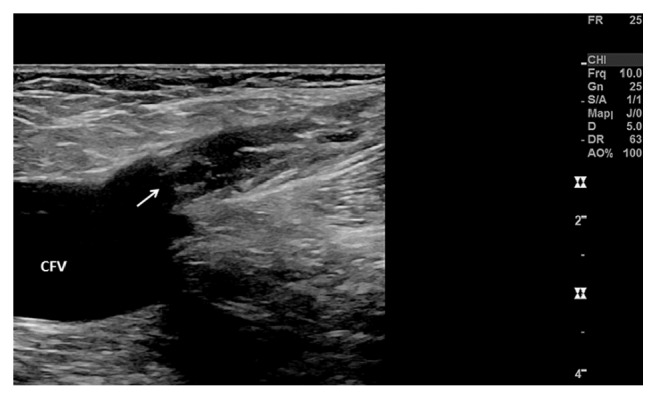
Result 13 days after endovenous laser ablation of the great saphenous vein (GSV) with exclusion of the thrombotic mass. The GSV (longitudinal view) is occluded up to the confluence (white arrow) between the great saphenous vein and the common femoral vein (CFV).

Duplex ultrasound visits on day 1 and day 13, at 6 weeks, and one year after the intervention showed a permanent occlusion of the treated GSV up to the confluence without evidence of any thrombus propagation into the deep vein system (Figure 4). Particularly, no residual mass could be detected at the level of the SFJ.

## 3. Discussion

Deep venous thrombosis and pulmonary embolism (VTE) are potential complications that arise in 8% of patients affected by superficial venous thrombosis (SVT), particularly if the thrombus extends to the SFJ [2]. The treatment options of varicose vein thrombosis include compression therapy, pain control, anticoagulation, and surgical or endovascular interventions such as sclerotherapy, high ligation, vein stripping, phlebectomy, and thermal ablation [1]. The lack of consensus regarding the best care of this condition reflects the limited existing evidence as resumed in a 2013 published Cochrane review [3].

In our case, we used an accelerated EVLA to prevent VTE (obviating the need for a prolonged anticoagulation) and to treat the varicosities. Thermal ablation of the GSV in patients with SVT distally from the SFJ was already performed with success and described by Enzler [4], Bishara [5], and Gradma [6]. However, in our case, the thrombotic mass was located directly at the SFJ and could be excluded from the circulation by bypassing it with the laser fiber and treating the involved segment. Dislocation of the thrombus during placement of the laser fiber poses a potential risk. As the patient was generally healthy, the thrombotic mass was relatively small, and the patient had been anticoagulated during 7 days, the risk of a cardiopulmonal compromising lung embolism was deemed to be low. Furthermore, we expected an experienced operator, using a high-resolution ultrasound probe (6-15 MHZ), to be able to navigate the fiber tip past the wall adherent, nonocclusive thrombus quite safely.

Two potential alternative treatment options were discussed. First, a traditional surgical exploration and high ligation of the SFJ would have been possible. However, the risk of an embolization of the thrombotic mass by manual manipulation would still have been present. Therefore, also considering the less invasive nature of endothermal ablation as compared to surgery [7], we decided to use the endovenous approach. Alternatively, a prolonged (therapeutic) anticoagulation with an ultrasound follow up after 4-8 weeks to check for thrombus resolution, could have been chosen. However, as the treatment of the varicose veins would have been required later on anyway, the patient opted for the immediate exclusion of the GSV.

## 4. Conclusion

EVLA may be an alternative treatment option to anticoagulation or conventional surgery in the case of a partially thrombosed varicose GSV in the proximity of the SFJ, obviating the need for a prolonged therapeutic anticoagulation or a surgical procedure.
